# Rhizosphere microbial community composition shifts diurnally and in response to natural variation in host clock phenotype

**DOI:** 10.1128/msystems.01487-21

**Published:** 2023-05-22

**Authors:** Charley J. Hubbard, Joshua G. Harrison, Robby McMinn, Julian C. Bennett Ponsford, Lois Maignien, Brent Ewers, Cynthia Weinig

**Affiliations:** 1 Department of Botany, University of Wyoming, Laramie, Wyoming, USA; 2 Program in Ecology, University of Wyoming, Laramie, Wyoming, USA; 3 Marine Biological Laboratory, Josephine Bay Paul Center, Woods Hole, Massachusetts, USA; 4 Laboratory of Microbiology of Extreme Environments, UMR 6197, Institut Européen de la Mer, Université de Bretagne Occidentale, Plouzane, France; 5 Department of Molecular Biology, University of Wyoming, Laramie, Wyoming, USA; USDA-Agricultural Research Service, Boyce Thompson Institute, Cornell University, Ithaca, New York, USA

**Keywords:** rhizosphere, diel cylcing, microbiome, dynamics

## Abstract

Plant-associated microbial assemblages are known to shift at time scales aligned with plant phenology, as influenced by the changes in plant-derived nutrient concentrations and abiotic conditions observed over a growing season. But these same factors can change dramatically in a sub-24-hour period, and it is poorly understood how such diel cycling may influence plant-associated microbiomes. Plants respond to the change from day to night via mechanisms collectively referred to as the internal “clock,” and clock phenotypes are associated with shifts in rhizosphere exudates and other changes that we hypothesize could affect rhizosphere microbes. The mustard *Boechera stricta* has wild populations that contain multiple clock phenotypes of either a 21- or a 24-hour cycle. We grew plants of both phenotypes (two genotypes per phenotype) in incubators that simulated natural diel cycling or that maintained constant light and temperature. Under both cycling and constant conditions, the extracted DNA concentration and the composition of rhizosphere microbial assemblages differed between time points, with daytime DNA concentrations often triple what were observed at night and microbial community composition differing by, for instance, up to 17%. While we found that plants of different genotypes were associated with variation in rhizosphere assemblages, we did not see an effect on soil conditioned by a particular host plant circadian phenotype on subsequent generations of plants. Our results suggest that rhizosphere microbiomes are dynamic at sub-24-hour periods, and those dynamics are shaped by diel cycling in host plant phenotype.

**IMPORTANCE:**

We find that the rhizosphere microbiome shifts in composition and extractable DNA concentration in sub-24-hour periods as influenced by the plant host’s internal clock. These results suggest that host plant clock phenotypes could be an important determinant of variation in rhizosphere microbiomes.

## INTRODUCTION

Plants can influence the assembly of microbial communities in the rhizosphere zone immediately surrounding their roots by influencing the supply of carbon, water, and other nutrients (1 - 3). Over the lifetime of a plant, the composition of rhizosphere microbial communities shifts in response to developmentally programmed changes in plant physiology (4 - 6). For instance, Chaparro et al. (6) observed variation in rhizosphere bacterial communities corresponding to changes in exudation during plant development. Although rhizosphere community assembly dynamics are comparatively well described on long temporal scales, such as over the life of a plant host and across growing seasons, the extent to which rhizosphere microbial communities shift on shorter, diurnal scales has only recently been considered (7 - 9), and little is known regarding the biotic and abiotic factors that could govern this cycling.

Research conducted to date exploring how plants influence their microbiomes has generally taken a candidate gene approach and used experimental genetic materials, such as knockout mutants (10). The design of these studies allows for the characterization of the effects on microbial community composition of the mutant genotype vs the cognate wild type, with ensuing implications for how a specific host genetic pathway affects the plant microbiome. For example, Hubbard et al. (7) used *Arabidopsis* to demonstrate that single gene mutations eliciting circadian clock periods shorter or longer than wild-type values significantly altered rhizosphere microbial community composition and function. Studies using mutants are effective at describing the effect that a functional vs. non-functional host pathway has on the associated microbial community but do not reveal how naturally occurring allelic variation in host pathways affects microbial communities. Thus, translational studies using wild genotypes are an important complement to knockout studies in model systems.

Through shaping the composition of associated microbial communities, individual plant hosts may also affect microbiome function with consequences for following generations of plants (11). For instance, several studies have shown that microbial communities shaped by differences in host plant genotype in one generation differentially affect plant performance in a second generation (7, 12, 13). As an example, Zolla et al. (13) collected rhizosphere soil from diverse wild and crop species, including *Arabidopsis thaliana*, and then used the conditioned soils to inoculate a second generation of *A. thaliana* plants. In the second generation, there was a significant variation in plant biomass between the compositionally distinct microbiome treatments. Thus, phenotypic variation among host plants may lead to differences in rhizosphere community composition and, as a result, differences in rhizosphere community function, as measured by plant performance.

In this study, we ask if diurnal shifts in composition occur within the rhizosphere bacterial community of *Boechera stricta*, a wild Brassicaceous relative of *A. thaliana* native to western montane regions of North America. We hypothesize that rhizosphere microbial abundance and community composition will shift between day and night time points in response to diurnal changes in host plant physiology (or potentially in direct response to changes in temperature and light). Additionally, we anticipate that some microbial taxa may be directly affected by clock-mediated physiology, which would be manifested as differential abundance between subjective day and night time points under free-running conditions (i.e., conditions that have constant temperature and light), as well as cycling conditions (diurnally changing light and temperature). We further hypothesize that rhizosphere community composition will be affected by plant circadian period or clock phenotype. That is, we anticipate that physiological differences brought about by match (or mismatch) between endogenous plant and environmental cycles will lead to differences in rhizosphere communities between plants with shorter (21-hour) and longer (24-hour) clock phenotypes. Finally, if clock phenotype alters rhizosphere community composition in one generation, then we ask if such differences may bring about differences in plant performance in a second generation of plants inoculated with the selected microbial communities.

## MATERIALS AND METHODS

### Plant material and growth conditions

To test the influence of the circadian clock on rhizosphere bacterial community assembly, we used four *Boechera stricta* genotypes from a population located near South Brush Creek within the Snowy Mountains of Wyoming (41.325°N, −106.502°W). The South Brush Creek population is known to harbor genetic variation in the circadian period, and the four inbred genotypes used here reflect extreme circadian period values: genotype 4 (circadian period ~24 hours), genotype 6 (circadian period ~24 hours), genotype 15 (circadian period ~21 hours), and genotype 20 (circadian period ~21 hours) (14). The preceding circadian period phenotypes described by Salmela et al. (14) were confirmed by McMinn et al. (15), who also found a minimum clock period of ~21 hours and a maximum of ~24 hours in the South Brush Creek population. As described in Methods below, the current study uses the same entrainment and free-running conditions as used by Salmela et al. (14); hence experimental genotypes can be expected to express the same 21- and 24-hour clock phenotypes in the current study.

Seeds were surface sterilized using a 10% bleach solution and placed in pots containing a mixture of sterilized Redi Earth potting mix (Sun Gro, Agawam, MA, USA) and soil inoculum. To create the soil inoculum, 70 g of South Brush Creek soil was mixed with 630 mL of reverse osmosis–filtered water and filtered through 1,000 µL, 500 µL, and 212 µL sieve to remove soil nematodes that could affect plant performance (16). For cycling conditions, plants were grown in Percival PGC-9/2 growth chamber (Percival Scientific, Perry, IN, USA), which was programmed to simulate fall conditions for entrainment, including a 15-hour photoperiod and diel temperature shifts from 4.9°C to 17.1°C and resembling conditions used in Salmela et al. (14) (Table S1). For free-running conditions, plants were grown under constant light and temperature (16.1°C). Under all conditions, photosynthetic photon flux density was 350 µmol photons/m^2^/s.

### Experimental design

#### Experiment 1: diurnal shifts in rhizosphere microbial community composition and extractable DNA

To characterize changes in rhizosphere communities between day and night time points, replicates of genotype 6 (~24-hour cycle) and genotype 15 (~21-hour cycle) were grown in a fully randomized design in simulated fall seasonal conditions for 5 weeks. Replicates were moved to either (i) simulated fall conditions or (ii) free-running conditions with constant light and temperature. Any observed changes in microbial community composition under fall conditions, which cycle in temperature and light availability, reflect either the direct microbial responses to daily changes in these factors or the indirect effects of daily fluctuations in plant host physiology. Any cyclic changes in microbial community composition observed under free-running conditions would reflect the effects of the endogenous plant host clock only since temperature and light were held constant for this treatment. For this experiment, 7 replicates of genotypes 6 and 15 growing in cycling conditions were destructively harvested over each of 3 days at 7 p.m. (2 hours before dark) and 4 a.m. (2 hours before light) (*n* = 84). Replicates (7 each of genotypes 6 and 15) growing in free-running conditions were harvested once, at 7 p.m. and 4 a.m. on the last day of the study, after growing for 3 days (*n* = 28). Samples were stored at −80°C prior to further processing.

#### Experiment 2: rhizosphere community response to clock phenotype of the plant host

To describe the influence of host clock phenotype on rhizosphere bacterial community assembly, replicates of genotypes 4 (~24-hour cycle), 6 (~24-hour cycle), 15 (~21-hour cycle), and 20 (~21-hour cycle) were grown in a fully randomized design in simulated fall conditions for 5 weeks at which point seven replicates of each genotype were destructively harvested for rhizosphere samples (*n* = 28). In addition, three replicates of rhizosphere soil from each genotype were collected for use in Experiment 3. Rhizosphere samples were stored at −80°C prior to further processing, while soil samples were used immediately (within 1 day) in Experiment 3.

#### Experiment 3: rhizosphere community feedback on plant performance

To assess the effects of rhizosphere communities shaped by host clock phenotypes on plant performance, replicates of genotypes 6 and 15 were grown in sterilized potting mix inoculated with soil slurries generated by genotypes 4, 6, 15, and 20 that were collected in Experiment 2 (*n* = 160; 2 genotypes × 4 inoculates × 20 replicates). After 6 weeks of growth, plants were harvested and aboveground and belowground biomass were measured.

### DNA extractions and amplicon sequencing

To extract rhizosphere bacterial DNA, plant roots were shaken to remove loosely adhering soil particles and then agitated in 3 mL of phosphate-buffered saline (PBS) to separate plant roots from rhizosphere soil particles. After 10 min, plant roots were removed with sterile forceps, and the soil and PBS solution were centrifuged at 3,000 relative centrifugal field (RCF) for 10 min as described in Hubbard et al. (7). The supernatant was discarded and ~250 mg of the pellet was placed into a bead tube from the Mobio Power Soil DNA Isolation Kit (Mobio Laboratories, Carlsbad, CA, USA). DNA was extracted following the manufacturer’s instructions, and soilless blanks were included in each round of extraction to ensure reagent sterility. To quantify DNA yields, we used an Invitrogen Qubit 4 (Thermo Fisher Scientific, Waltham, MA, USA). DNA yields were then divided by the pellet mass to obtain a rough approximation of DNA concentration.

Samples were sent to the Marine Biological Laboratories (Woods Hole, MA, USA) for amplicon library preparation of the V4–5 region of the 16s ribosomal subunit gene (518F and 926R) for amplicon sequencing on the Illumina MiSeq platform (Illumina, San Diego, CA, USA) as described in Newton et al. (17). We used the R package *dada2* (using default parameters) to convert raw reads into amplicon sequence variants (ASVs) and taxonomy tables by filtering reads based on the quality, removing primer-binding regions, inferring sequencing error rates, merging paired-end reads, removing chimeras, and assigning taxonomy based on the Silva reference database (version 128) (17 - 20).

### Rhizosphere community and statistical analyses

To characterize the influence of the host plant clock on rhizosphere community composition, we used the R packages *phyloseq* and *vegan* (20, 21). We used permutational multivariate analysis of variance (ANOVA) using distance matrices (the adonis function) on Bray–Curtis dissimilarities to describe broad changes in rhizosphere community composition attributable to time point or host plant clock phenotype (23). Our model was of the form: Dissimilarity = experimental day (time point) + time point, where experimental day nested within time point tested for potential differences in microbial communities between collections performed on the first, second, and third day and time point tested for differences between day and night. To characterize the effects of clock phenotype, we used a model with the form: Dissimilarity = genotype (clock phenotype) + clock phenotype, where the first nested term tested for differences between genotypes within a clock phenotypic class and clock phenotype tested the effect of host circadian period (21 and 24 hours). This analysis characterizes coarse-grained changes in rhizosphere community composition (as influenced by the presence vs absence and abundance of all taxa within the community); therefore, we paired this analysis with Dirichlet-multinomial modelling (DMM) to identify differentially abundant ASVs between time points and clock genotypes (Experiment 2) (24). DMM estimates the proportional relative abundances of taxa within each sample as the parameters of multinomial distributions. The prior for these multinomial distributions is a Dirichlet distribution, with parameters that represent the proportional relative abundance of microbial taxa within a sampling group. This method has several benefits, including sensitive detection of taxa that differ among treatment groups and the sharing of information among replicates within a treatment group via the model’s hierarchical structure (24). Additionally, we used DMM to determine the effect of free-running vs cycling conditions for genotypes 6 and 15 to identify microbes that could be responding to plant diel physiology (Experiment 1). We also looked for overlap in ASVs within and between short- and long-period clock genotypes that were associated with the effect of clock genotype on rhizosphere community composition or the differences in rhizosphere community function in the context of plant performance (Experiment 3).

We used two-way ANOVAs to characterize the influence of soil microbiome treatment and plant genotype on aboveground biomass to determine if overstory history influenced plant performance.

## RESULTS

### Experiment 1: rhizosphere microbial community composition and extractable DNA shifts diurnally

**Fig 1:**
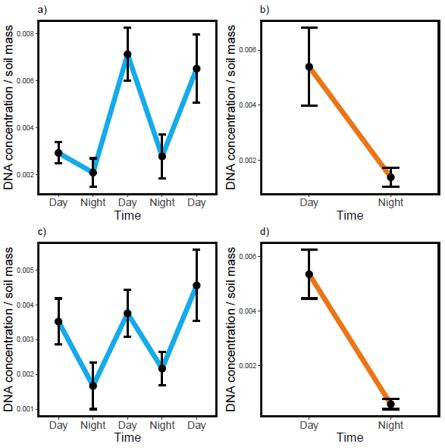
Concentration of extracted DNA as a function of sampling time. DNA concentration (ng/μL; 16s locus) was divided by soil mass (milligram) to estimate microbial biomass within samples and is shown on the *y*-axis (see Discussion for caveats to this approach). Sampling time is shown on the *x*-axis. Panels (a) and (b) show DNA concentration for genotype 6 in cycling (a) and free-running conditions (b). Likewise, panels (c) and (d) show DNA concentration for genotype 15 in cycling (c) and free-running (d) conditions. Note that time in (b) and (d) is subjective, that is, day and night reflect the times that light and dark periods would have been experienced by the experimental plants based on the preceding cycling conditions. Bars show 95% confidence intervals around estimates.

**Fig 2:**
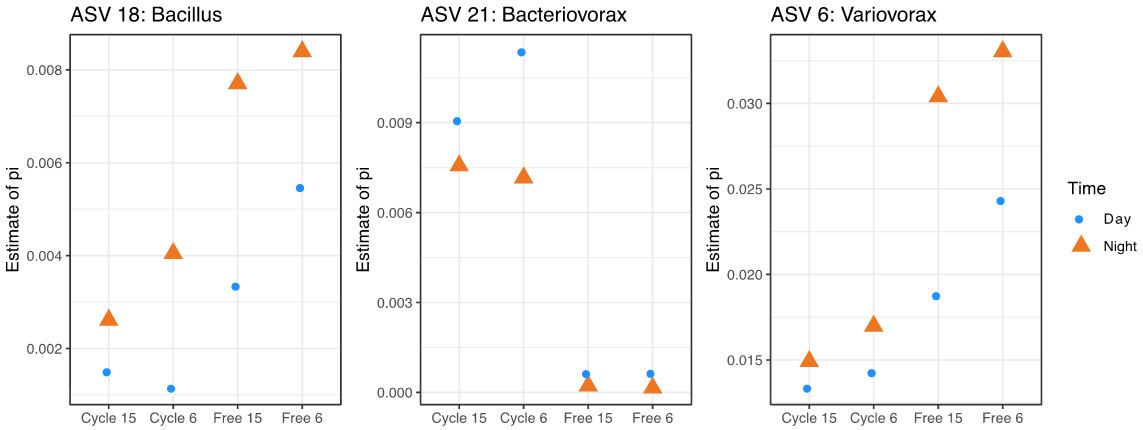
Differences in relative abundances of several ASVs that were associated with day and night time points. Relative abundances are shown on the *y*-axis as estimates of Dirichlet pi parameters (sampling group-wide proportions). Treatment, either cycling or free-running, and genotype are shown on the *x*-axis. Colors of points denote sampling time. DMM confirmed a highly certain difference in relative abundances for these taxa between night and day time points.

The DNA concentration obtained from the rhizosphere changed significantly between day and night time points, as measured per gram of soil (repeated measures ANOVA; *P* < 0.001; Fig. 1). In both cycling and free-running conditions and across genotypes, bacterial DNA concentration was larger during the day than at night. We also observed that rhizosphere community composition changed significantly between day and night time points. In cycling conditions, 4.7% and 3.4% of ASVs were differentially abundant between time points for genotypes 6 and 15, respectively, as determined by DMM. Similarly, in free-running conditions, 15.5% and 17.3% of ASVs were differentially abundant between time points for genotypes 6 and 15, respectively. Likewise, permutational multivariate analysis of variance (PERMANOVA) on Bray–Curtis dissimilarities revealed significant differences in bacterial community composition between day and night time points in free-running (*P* = 0.001; R^2^ = 0.13) and cycling conditions (*P* = 0.001; R^2^ = 0.28). In total, we found three ASVs (*Bacillus*, *Bacteriovorax*, and *Variovorax*) that were (i) identified as differentially abundant through DMM and (ii) consistently associated with day or night time points across environmental treatments and genotypes (Fig. 2). Fifty-one ASVs were differentially abundant between short-period and long-period genotypes in free-running conditions, while 31 ASVs were differentially abundant between those genotypes in cycling conditions.

### Experiment 2: rhizosphere communities are influenced by the clock phenotype of plant hosts

Rhizosphere bacterial community composition was strongly affected by host clock phenotype (Fig. S1 and S2). PERMANOVA on Bray–Curtis dissimilarities revealed significant differences between plants expressing short (21-hour) and long (24-hour) clock phenotypes (*P* = 0.023). DMM identified 15.9% of ASVs differentially abundant between genotypes 6 and 15, 10.9% between genotypes 6 and 20, 15.3% between genotypes 4 and 15, and 19.2% between genotypes 4 and 20. Furthermore, seven ASVs (*Sphingobium*, *Flavobacterium*, *Chitinophagaceae*, *Dyadobacter*, *Phenylobacterium*, *Verrucomicrobia*, *Undibacterium*) were associated with short-period genotypes across comparisons, while four ASVs (*Massilia*, *Variovorax*, *Devosia*, *Pedobacter*) were associated with long-period genotypes across comparisons.

### Experiment 3: rhizosphere community feedback on plant performance

**Fig 3:**
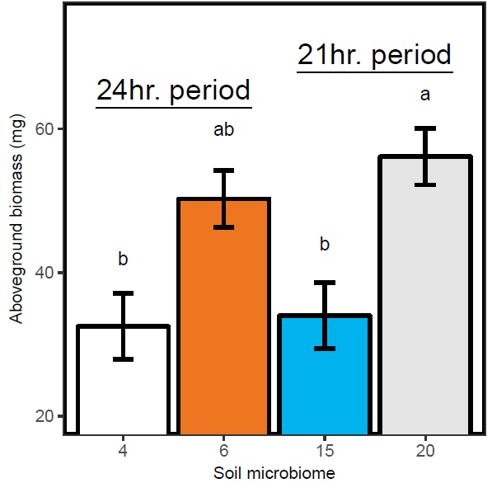
Plant biomass as a function of rhizosphere microbiome conditioning via overstory history. For this experiment, plants of genotypes 6 and 15 were grown in soil that had been previously used to grow plants of a particular genotype (shown on the *x*-axis). Since each genotype influences the rhizosphere microbiome uniquely, this experiment tested if the conditioning of a particular genotype generally led to increased performance in subsequent generations. Genotypes differed in that they had short (21-hour) or long (24-hour) period clock phenotypes (as labeled). We observed plants of genotype 20 were larger than genotypes 4 and 15, but we did not see any consistent effects associated with clock phenotype nor did we observe treatment by genotype interactions (*P* = 0.71).

Overstory history, in terms of clock phenotype, did not have a notable effect on plant growth promotion (Fig. 3; *P* = 0.894). However, overstory history in terms of genotype did have an effect on plant performance (*P* = 0.002), where plants grown in soils with an overstory history of genotype 20 were significantly larger than plants grown in soils with a history of genotype 4 (*P* = 0.034) or 15 (*P* = 0.021).

## DISCUSSION

Until recently, the temporal scales in which rhizosphere microbial communities change in response to plant physiology were thought to be primarily on the order of months or years (5, 6). Our results suggest that plants can have a much more immediate effect on associated microbes. Indeed, the effect of sampling time on DNA concentration, a proxy for microbial assemblage biomass (see below), was quite notable—in some cases, we observed a tripling or near-quadrupling of DNA from night to day (Fig. 1). Microbial composition also shifted significantly between day and night time points (Fig. S1 and S2). We found that time points explained ~13% of the variation in coarse-grained community composition (estimated from Bray–Curtis dissimilarity analyses) and up to 17.3% of the variation in individual ASV abundance (estimated by DMM). These results add to a small but growing body of work demonstrating rhizosphere microbial assembly dynamics within sub-24-hour time scales (7 - 9).

The mechanisms associated with the differences in rhizosphere assemblages between day and night are unknown but could reflect various diurnal physiological processes in the host plant, including changes in transpiration and exudation. During the day, water transport from the soil to leaves is highest, while at night, stomata close, reducing the demand for water (3). As a result, microbes may be physically transported into and out of the rhizosphere. Root exudation is also diurnally patterned (24 - 28) and may also explain compositional differences between the time points, at least in part. Numerous other phenotypic changes in the plant host (and perhaps in the microbes themselves) occur with a regularity defined by day length and parsing the relative influence of these plant traits on the rhizosphere will be a profitable line of inquiry.

We used DNA concentration as a proxy for bacterial biomass in the rhizosphere rather than cell counts, which would have been impractical to obtain. Previous work has shown that extracted DNA concentration is correlated with microbial cell counts in soils (29) and water (29, 30). We used the same extraction technique for all samples and processed all samples together, thus minimizing the possibility of batch effects. We hypothesize that variation in extraction difficulty between time points does not underlie our results, given that the substrates extracted were very similar to one another and were expected to differ only in rhizosphere microbiota. Microbial taxa do differ in how easy it is to extract DNA from their tissues, thus the shifts in microbial composition that we observed from day to night could have influenced the diel cycling of extractable DNA. A caveat to our results is that DNA from the plant could be a component of total DNA concentration. If host plants shed more DNA during the day than at night, and that DNA is catabolized prior to night time sampling, then this could underlie the dramatic sub-24-hour shifts in concentration that we observed. If this hypothesis were true, then the breakdown of plant DNA could be an important nutrient source for rhizosphere microbes.

We also compared the extent to which abiotic environmental factors (changes in light and temperature) vs clock phenotype influenced rhizosphere dynamics. We found consistent changes in the size and composition of rhizosphere communities between day and night time points among plants growing not only in cycling but also in free-running conditions (Fig. 1), suggesting a primacy of clock phenotype in the results that we observed. In particular, microbial community size increased under subjective day in a manner that reiterated patterns observed under true day time points of cycling conditions. Alone, the endogenous clock affected the assembly dynamics of large percentages of the rhizosphere bacterial microbiome (15.5%–17.3% of all differentially abundant ASVs in free-running conditions). Furthermore, we identified two ASVs (*Bacillus* and *Variovorax*) that were differentially abundant and associated with night time points and one ASV (*Bacteriovorax*) associated with day time points across clock genotypes and experimental conditions. It is possible that these microbes are cycling independently of the effects of the plant clock, as other organisms are known to possess circadian clocks (31 - 34). However, because these bacteria are consistently associated with specific time points across plant genotypes and environmental conditions, they seemingly have intimate associations with host physiology regulated by the clock and may be targeted to better understand plant–microbe interactions on a molecular level.

Recent work in *A. thaliana* has shown that knockout mutations in the clock gene, *TIMING OF CAB EXPRESSION 1*, which shortens the circadian period from 24 to 20 hours, significantly altered rhizosphere bacterial community composition. Studies of knockout mutants are effective at characterizing the overall effect of specific genetic pathways on plant phenotypes (including the extended phenotype of the rhizosphere community) but cannot describe the effects of natural genetic variation found in wild populations. We thus characterized the rhizosphere communities associated with co-occurring *B. stricta* genotypes with naturally segregating short circadian periods (21 hours) vs longer circadian periods (24 hours) that respectively approximate the short-period *toc1* and wild-type genotypes of *A. thaliana*. We found significant differences in rhizosphere bacterial communities associated with plants with short vs long circadian periods and notably, that genotypes with short circadian cycles hosted less diverse rhizosphere microbial communities, as was the case for *Arabidopsis* short-period mutants (7). Differences in rhizosphere communities may reflect the mismatch between endogenous and environmental periods, where a mismatch leads to significant decrements in physiological traits, such as stomatal conductance, assimilation, and starch utilization that are important upstream determinants of root exudation (31 - 34). Notably, a short-period (21 hours) phenotype is anticipated to be more functionally severe and have stronger fitness consequences for plants, because long circadian periods still allow for accurate phase matching to dawn (34 - 37). Plants with circadian periods shorter than 24 hours that have reduced physiological function may serve as less favorable microbial hosts, thereby accounting for the reduced diversity of microbial communities found with short-period genotypes of both *A. thaliana* and *B. stricta*.

As in any ecosystem, community composition is related to function (11, 37). However, unlike in an earlier study, where plants grown in soils with an overstory history of a 24-hour clock phenotype outperformed plants grown in soils with an overstory history of short (20-hour) and long (28-hour) period mutants, we did not observe any significant differences in plant performance between plants grown in soils with a history of short (21-hour) or long (24-hour) period clock phenotypes (Fig. 3). Instead, we found that plants grown in soils with a history of genotype 20 were significantly larger than plants grown in soils with an overstory history of other genotypes. The growth-promoting nature of the genotype 20 microbiome may reflect natural variation in non-clock pathways that contribute to the shaping of beneficial rhizosphere communities. Genotype 20 had the highest root/shoot ratio of the genotypes used in the current study and differed from the other genotypes in microbial community composition (as evidenced by the significant effect of genotype nested within clock phenotype). Thus, while host clock phenotype significantly affected microbial community composition in the current study, the effect of a naturally occurring short circadian period may be less than that induced by a knockout mutation in the host clock (7). As a result, other plant functional traits could be playing a more important role in the assembly of beneficial microbial communities, including relative host allocation to roots and the physical environment of the microbes.

In sum, in combination with studies showing altered rhizosphere community composition in *A. thaliana* clock mutants with modified clock timing (7, 8), our results using genotypes with naturally segregating clock variation suggest that the plant circadian clock should be regarded as a key regulator of rhizosphere assembly dynamics. The extent to which the host circadian clock may shape the rhizosphere microbiome to its advantage requires further research. While Hubbard et al. (7) observed that a wild-type clock phenotype conditioned the soil microbiome in a manner that improved plant growth, this pattern was not observed in the current study. Future work should also explore the mechanisms by which plant clocks exert their influence and if that influence could extend to microbes living within other plant compartments.

## Data Availability

Raw reads were deposited into the Sequence Read Archive under PRJNA857872. Additional data, including concentration of DNA extracted per sample, sequence counts, and plant performance metrics, are available at: https://doi.org/10.15786/16968028.v1.

## SUPPLEMENTAL MATERIAL

FIG S1 msystems.01487-21-s0001.pdf Principal coordinate analysis of a Hellinger distance matrix of microbial sequence count data for plants reared under free-running conditions (for details, see main text).

FIG S2 msystems.01487-21-s0002.pdf Principal coordinate analysis of a Hellinger distance matrix of microbial sequence count data for plants reared under cycling conditions (for details, see main text). Time points alternate in terms of day and night conditions.

TABLE S1 msystems.01487-21-s0003.docx Fall conditions used in Experiments 1 and 2 described in Salmela et al. (14). Lights were on between 6 a.m. and 9 p.m.

ASM does not own the copyrights to Supplemental Material that may be linked to, or accessed through, an article. The authors have granted ASM a non-exclusive, world-wide license to publish the Supplemental Material files. Please contact the corresponding author directly for reuse.
