# Supplementary figures and images for: Rhizosphere microbial community composition shifts diurnally and in response to natural variation in host clock phenotype
Source: mSystems. 2023 May 22;8(3):e01487-21. doi: 10.1128/msystems.01487-21 (PMC10308896; doi:10.1128/msystems.01487-21)

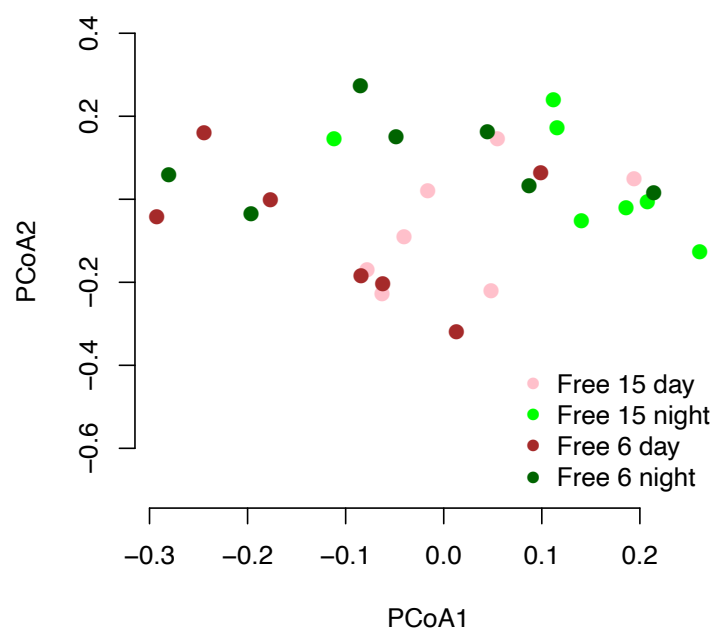

Supplement: FIG S1 — Principal coordinate analysis of a Hellinger distance matrix of microbial sequence count data for plants reared under free-running conditions (for details, see main text). [file msystems.01487-21-s0001.pdf]

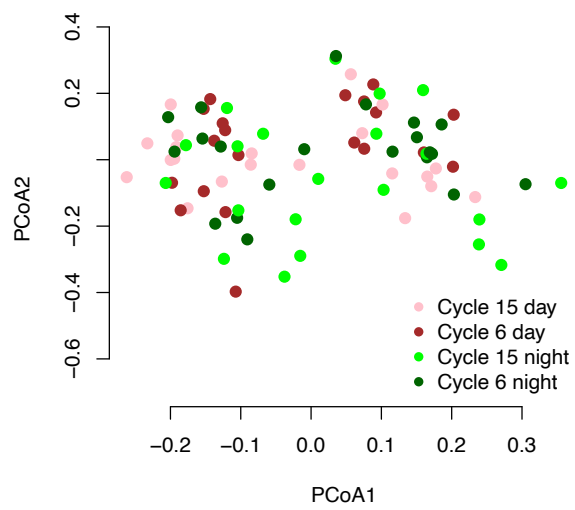

Supplement: FIG S2 — Principal coordinate analysis of a Hellinger distance matrix of microbial sequence count data for plants reared under cycling conditions (for details, see main text). Time points alternate in terms of day and night conditions. [file msystems.01487-21-s0002.pdf]
