# Supplementary material for: Rhizosphere microbial community composition shifts diurnally and in response to natural variation in host clock phenotype
Source: mSystems. 2023 May 22;8(3):e01487-21. doi: 10.1128/msystems.01487-21 (PMC10308896; doi:10.1128/msystems.01487-21)
Supplement: TABLE S1 — Fall conditions used in Experiments 1 and 2 described in Salmela et al. (14). Lights were on between 6 a.m. and 9 p.m. [file msystems.01487-21-s0003.docx]

**Supplemental Table 1**: Fall conditions used in *Experiments 1* and *2* described in Salmela *et al*. (2016). Lights were on between 6 A.M. and 9 P.M.

| **Time** | **Temperature** | **Time** | **Temperature** |
| --- | --- | --- | --- |
| 12 A.M. | 6.2 | 12 P.M. | 16.1 |
| 1 | 5.8 | 1 | 16.9 |
| 2 | 5.4 | 2 | 16.9 |
| 3 | 5.1 | 3 | 17.1 |
| 4 | 4.9 | 4 | 15.5 |
| 5 | 7.3 | 5 | 14.7 |
| 6 | 10 | 6 | 12.3 |
| 7 | 12.6 | 7 | 10.6 |
| 8 | 14.2 | 8 | 9 |
| 9 | 15.3 | 9 | 8.4 |
| 10 | 16.5 | 10 | 8.2 |
| 11 | 17 | 11 | 7.7 |
